# Predicting Cannabis Abuse Screening Test (CAST) Scores: A Recursive Partitioning Analysis Using Survey Data from Czech Republic, Italy, the Netherlands and Sweden

**DOI:** 10.1371/journal.pone.0108298

**Published:** 2014-09-29

**Authors:** Matthijs Blankers, Tom Frijns, Vendula Belackova, Carla Rossi, Bengt Svensson, Franz Trautmann, Margriet van Laar

**Affiliations:** 1 Department of Drug Monitoring, Trimbos Institute, Utrecht, the Netherlands; 2 Department of Psychiatry, Academic Medical Centre, University of Amsterdam, Amsterdam, the Netherlands; 3 Department of Research, Arkin, Amsterdam, the Netherlands; 4 Department of Addictology, First Faculty of Medicine, Charles University and General University Hospital, Prague, Czech Republic; 5 Centre for Biostatistics and Bioinformatics, University Rome Tor Vergata, Rome, Italy; 6 Department of Social Work, Malmö University, Malmö, Sweden; University of Insubria, Italy

## Abstract

**Introduction:**

Cannabis is Europe's most commonly used illicit drug. Some users do not develop dependence or other problems, whereas others do. Many factors are associated with the occurrence of cannabis-related disorders. This makes it difficult to identify key risk factors and markers to profile at-risk cannabis users using traditional hypothesis-driven approaches. Therefore, the use of a data-mining technique called binary recursive partitioning is demonstrated in this study by creating a classification tree to profile at-risk users.

**Methods:**

59 variables on cannabis use and drug market experiences were extracted from an internet-based survey dataset collected in four European countries (Czech Republic, Italy, Netherlands and Sweden), n = 2617. These 59 potential predictors of problematic cannabis use were used to partition individual respondents into subgroups with low and high risk of having a cannabis use disorder, based on their responses on the Cannabis Abuse Screening Test. Both a generic model for the four countries combined and four country-specific models were constructed.

**Results:**

Of the 59 variables included in the first analysis step, only three variables were required to construct a generic partitioning model to classify high risk cannabis users with 65–73% accuracy. Based on the generic model for the four countries combined, the highest risk for cannabis use disorder is seen in participants reporting a cannabis use on more than 200 days in the last 12 months. In comparison to the generic model, the country-specific models led to modest, non-significant improvements in classification accuracy, with an exception for Italy (p = 0.01).

**Conclusion:**

Using recursive partitioning, it is feasible to construct classification trees based on only a few variables with acceptable performance to classify cannabis users into groups with low or high risk of meeting criteria for cannabis use disorder. The number of cannabis use days in the last 12 months is the most relevant variable. The identified variables may be considered for use in future screeners for cannabis use disorders.

## Introduction

Cannabis is Europe's most commonly used illicit drug with approximately 20 million adults having used the drug in the last year, which is about 6% of the population aged 15–64 years [1]. An indication of the public health impact of cannabis use is reflected in data on patients entering specialized treatment in Europe for substance use disorders: Cannabis is the second most frequently reported substance, after heroin [1]. At the same time, many users of cannabis do not develop substance use disorders or other problems associated with their cannabis use [2], [3]. Patterns of substance use such as frequency of use or social contexts of use are important predictors of substance use disorders [4], [5]. In a recent study, living alone, coping motives for cannabis use, recent negative life events, and cannabis use disorder symptoms were found to predict first incidence of cannabis dependence [2].

Against this backdrop, two important public health challenges can be raised: (1) to develop, validate and implement screening tools to identify those at-risk to develop cannabis use disorders, and (2) to identify patterns of risk factors or markers associated with at-risk use, in order to target drug policy efforts to individuals manifesting these patterns [53]. Recently, a number of screening instruments for risky substance use have been developed and validated for cannabis, including the Severity of Dependence Scale (SDS) [6], [7], the Cannabis Use Disorder Identification Test (CUDIT) [8], [9], and the Cannabis Abuse Screening Test (CAST) [10]. Annaheim [11] provides a recent review in which she identified 44 potentially useful cannabis problems screening tools. She found the CAST to be among three of the most appropriate instruments [11].

Since 2000, a large number of publications on predictors for cannabis dependence and risk factors associated with cannabis-related problems have been published. Using the Medline search string [(cannabi* OR marijuana OR marihuana OR weed OR hash) AND (“risk factor*” OR “predict*” OR associat*) AND (harm OR problem* OR dependenc* OR abus*)], 3187 publications between January 1, 2000 and March 1, 2014 were identified, including 382 review articles. Among the associations covered in these publications are those between cannabis and genetic and/or environmental factors [17], stress [18], other mental health disorders [19] including juvenile psychiatric disorders [20]; the link between cannabis and psychosis/schizophrenia [21], [22], neurocognitive [23] and neuroanatomical [24] correlates of cannabis use, between cannabis and socioeconomic status [25], and early onset of cannabis use [26]. A number of studies specifically assessed associations between use quantity and cannabis abuse/dependence [27], [28]. Quantity has been shown to discriminate dependent and non-dependent users independently from frequency of use [29], [30] although this finding has not been consistently reported [31]. Assessment of other substance use, education, but also delinquency factors are found to be important in identifying individuals at risk for perseveration of cannabis use [32].

All in all, numerous factors are associated with cannabis use initiation, perseveration and cannabis-related disorders. Therefore it is difficult to identify the characteristics of users at risk with traditional hypothesis-driven approaches. This challenge is not limited to profiling cannabis users. This is one of the reasons that the use of data mining approaches are becoming more widely used in the analysis of healthcare data (eg [33], [34]). However, their use in the field of problematic substance use remains limited [35]–[37]. The main difference between data mining approaches and more traditional hypothesis-based epidemiological approaches is that using data mining, patterns of association between (multiple) predictors and dependent(s) are explored without a priori hypotheses regarding these associations – provided that enough data are available [38]. Besides the prerequisite of sufficiently large datasets [39], [40], a potential risk of data mining is overfitting: modelling minor and random fluctuations in the data as if these are true effects. In order to avoid overfitting, it is necessary to use cross-validation procedures: testing the model's ability to generalize by evaluating its performance on a set of data not used for model development [41].

In this study, the use of a data-mining approach called binary recursive partitioning, utilized to generate classification and regression trees (CART) is demonstrated. Recursive partitioning can be used to identify variables that are of relevance to the outcome of interest, but also to create CARTs [35]. Recursive partitioning is a non-parametric regression approach; its main characteristic is that the space spanned by all predictor variables is recursively partitioned into a set of areas. A partition is created such that observations with similar response values (dichotomized CAST scores in this study) are grouped together. After the partitioning is completed, a constant value of the response variable is predicted within each area [41]. As a result, recursive partitioning examines all available predictors and identifies a series of variables that are most related to the outcome measure. Zhang and Singer [45] have published an overview of recursive partitioning methods, classification trees, and applications. Examples of the use of recursive partitioning in addiction research include a study by Swan and colleagues [43], who identified relevant variables when examining the heterogeneity of their outcomes from a smoking cessation intervention using recursive partitioning. Others have for example used recursive partitioning in an analysis of pregnant women's responses to substance use questions, which resulted in a three-item Substance Use Risk Profile-Pregnancy scale [36]. In the current study, this technique will be applied to a pre-existing dataset comprising many potential predictors of cannabis use disorders, including social, epidemiological and drug use variables (demographics, cannabis use history, current use, use of other illegal substances), and cannabis market-related characteristics (methods of acquisition of cannabis and occurrences of police interference).

## Methods

### Ethics statement

The source of the data for the present analysis comes from EC project “Further insights into aspects of the illicit EU drugs market”, that included an internet-based survey performed in seven European member states [42]. For this analysis, data from the four (Czech Republic, Italy, the Netherlands and Sweden) of the seven countries are used. These four countries were selected based on the number of survey participants per country. The aim was to maximise the number of respondents per country, in order to develop country-specific models later on in the study. The survey has been conducted in compliance with the Helsinki Declaration. The original study was approved by the Medical ethics Committee of the University Medical Centre Utrecht, the Netherlands. Approval from a Medical ethics Committee was not deemed necessary in the other participating countries. All participants provided informed consent online and were provided with contact information of the collaborating research centres.

### Recruitment of the survey sample

Recruitment in the four countries was managed online between February 6, 2012 and April 22, 2012. The target population comprised people who had used illegal drugs during the previous 12 months. Advertisements were posted on drug information websites and other drug related websites, web forums, newsletters and social media. For the current analysis, data from respondents who reported they had used cannabis in the 12 months before the survey were selected.

In Czech Republic, a Facebook page was created for the survey (www.facebook.com/drogy2012). This Facebook advertisement was presented to 129,160 people. The page was updated several times with articles related to drug policy. Moreover, an advertisement to encourage young adults (age 15–34) to participate was placed on Facebook. Invitations to participate in the study were distributed through websites focussing on dance events, cannabis-related topics, drug counselling sites (eg Adiktologie.cz) and through a discussion board on drug issues (nyx.cz) [42].

In Italy, promotional emails were sent through the mailing list of illicitdrugmarket.net, to various drug-related associations, and to 30,000 students of the University of Tor Vergata in Rome. Accounts and pages were created on Facebook, Twitter, and MySpace (Mercato della Droga). Announcements appeared in a blog of the Italian government broadcasting agency (RAI 3), and on the websites of several newspapers. Interviews with the principal researcher in Italy (author C. R.) were broadcasted on Radio Radicale (political radio) and on the national TV. In addition, leaflets were printed and distributed at rave parties, discos, soccer stadiums and other social gatherings [42].

In the Netherlands, online recruitment was facilitated by the peer-supported drug education and prevention network “Unity” through social media, and websites (eg partyflock.nl, a website targeted at adolescents frequenting rave parties) [42]. Offline recruitment using information leaflets was performed through the network of drug testing facilities and addiction treatment centres linked to the national Drug Information Monitoring System (DIMS), coordinated by the Trimbos Institute.

In Sweden, a text advertisement was placed on the website Flashback.org, an “underground” culture and lifestyle forum with around 130,000 daily page views. Advertisements were posted using Facebook and Twitter accounts. One of them was the Facebook group called “Centre for narcotics science” (Centrum för Narkotikavetenskap - CFN), a group that wants to push issues of harm reduction policies to the political agenda and could be defined as “drug liberal” within the Swedish context. An email with information on the survey was sent to all students of Malmö University [42].

### Survey procedure

The cannabis survey module was part of a larger survey study aimed at collecting data on consumption patterns and drug market characteristics perceived by (last year) users of cannabis, cocaine, ecstasy or (meth)amphetamine. This paper presents the data from participants of the cannabis survey. Survey instruments and questions were selected by a team of experts from the participating countries (see Acknowledgements). The web based survey was developed using Survey Monkey (www.surveymonkey.net), and tested by a small panel of experts and lay people for intelligibility, programming errors, estimated completion time, leading to a number of adjustments. The text of the resulting final survey was translated from English into each of the other countries languages by a native speaker. To ensure comparability, a sample of questions was back-translated. The web surveys were accessible for approximately 10 weeks [42].

Upon visiting the website, potential respondents were presented with a short introduction text, outlining the survey. This was followed by an informed consent form explaining the study and underlining the voluntary nature of participation, the anonymity of participants and the possibility to discontinue participation at any time without consequences. At the bottom of this page a choice between agreeing or declining to participate had to be indicated by clicking the corresponding button. After participants had given informed consent, they were asked for their gender and age, followed by questions regarding the last time they had used cannabis, cocaine, ecstasy or amphetamine in the last 12 months, in order to determine what survey they should be allocated to [42]. In case they reported to have used more than one of the substances, they were randomly allocated to one of the substances they indicated to have used. Survey responses were mandatory (participants could not proceed before these questions were answered, although a “I don't know” or equivalent answer option was available where applicable), except for questions on income.

### Measures

Items included in the survey were: cannabis use in the past 30 days and past 12 months, types of substances used, frequency of use, cannabis units (“joints”) consumed on a typical/last use day, route of administration, money spend on drugs, sources of supply, availability of other drugs at supply source, time and effort required to obtain drugs, and the CAST. The “availability of cannabis” section of the survey comprised of questions on purchasing or otherwise obtaining drugs (where, from who, ease and time needed to obtain) and on the availability of other drugs (degree of separation of the markets). This resulted in a total number of 59 variables. For reasons of comparability, questionnaires were harmonized for each country as much as possible, although some minor country-specific adjustments had to be made (eg some cannabis market questions in the Netherlands differed from those in countries with non-regulated cannabis markets) [42].

Dependent variable in the analyses was whether participants scored below or above a cut-off score of 7 on the full version of the CAST, a screening instrument for cannabis abuse among adolescents and young adults in general population surveys [10]. The CAST has been adopted as an optional module in the European School Survey Project on Alcohol and other Drugs (ESPAD) since 2007 [12]. More recently, the 6-item CAST has been validated in adolescent samples in France [5] and Italy [13] and Spain [14], [15] against diagnostic interviews and other screening instruments. Psychometric analyses indicate the CAST has good internal consistency and satisfactory concurrent validity, making it a brief and efficient instrument to identify at-risk adolescent cannabis users [5], [13], [14], [16]. Most analyses indicate that the CAST has a one-dimensional structure, although at least one study [14] found a two-dimensional factor structure. Other authors, for example Legleye and colleagues, have suggested a three-class solution with cut-offs of 3 and 7 for the medium/severe and severe classes respectively, using latent class analysis [5]. The cut-off score of <7 and ≥7 has been suggested by Cuenca-Royo and colleagues [14] to detect moderate and severe addiction (conform DSM-V [54]) with good reliability (AUC = 0.82), although a proxy to the DSM-V and not the DSM-V itself was used by Cuenca-Royo et al [14].

### Statistics

The full dataset for the four participating countries (n = 2617) was randomly split in a training set (approx. 80% of the cases, n = 2074) and a validation set (the other 20% of the cases, n = 543) for cross-validation of the classification trees. The classification trees were constructed using the training set, and the results were validated by applying the trees to the validation set. In order to construct a classification tree, a three step analytical approach was used. In step 1, using the data from the training set, all of the 59 potential predictors explored in this study were analysed using bivariate logistic regression, with the dichotomized CAST score equal to 7 or higher, or below 7 as the dependent variable. To account for multiple comparisons in the bivariate analysis step, Bonferroni correction was applied and α was set at 0.05/59 variables  = 0.001. Thus, only variables with a p-value ≤0.001 in the bivariate analysis step were selected as potential predictors for the multivariate logistic regression analysis in step 2. Here, variables with a significant association with the dichotomized CAST dependent variable were entered in the multivariate modelling procedure. This procedure implemented an iterative procedure to construct a multivariate main effects model with optimized goodness-of-fit (based on Nagelkerke R^2^) [44]. In step 3, the combination of variables that attained the optimal goodness-of-fit in the multivariate logistic regression analyses were entered in the recursive partitioning analysis. Recursive partitioning was performed using the computational recursive partitioning package “party” [46] version 1.0–8 for the R statistical environment version 3.0.1 [47]. The core of the package is an implementation of conditional inference trees which embed tree-structured regression models into a well-defined theory of conditional inference procedures. This nonparametric class of regression trees is applicable to all kinds of regression problems, including nominal, ordinal, numeric, censored as well as multivariate response variables and arbitrary measurement scales of the covariates [46]. For this analysis, the minimum criterion for making a split in the classification tree was set at α = 0.05, in accordance with the α-value in step 1 (before Bonferroni correction). In addition, Bonferroni correction was applied here as well. The minimum number of participants in a (terminal) node was set at n = 250 for the combined dataset (four countries), and at n = 75 for the separate country datasets. This meant that each split contained a substantial proportion (at least 9%) of all cases in order to reduce the risk of overfitting the data [48].

The training dataset (containing 80% of the data) was used to build a recursive partitioning model for the four countries combined. After the optimal model was constructed using the training data, performance statistics (accuracy, sensitivity, specificity, positive/negative predictive value) were calculated for the training set, the validation set, the full set, and for the four separate countries. Accuracy of each model was tested using a one-sided exact binomial test against the “no information rate” which is based on the prevalence rate of CAST score ≥7 in each dataset. In order to assess whether a country-specific model led to a significant improvement in classification accuracy compared to the combined model, country-specific recursive partitioning models were constructed, and their performance statistics were compared to those of the generic model using Pearson's chi-squared test (2-sided). All analysis in this study were performed using R statistical environment version 3.0.1 [47].

## Results

### Data cleaning, response rate and sample characteristics

In the data cleaning and restructuring step, erroneous responses were removed by controlling for multivariate outliers (based on Mahalanobis' Distance) and by removing participants who spent less than 7 minutes, or more than 45 minutes to complete the survey. Also, participants who did not complete the CAST section of the survey, or who did not provide responses on at least one of the other variables included in the analyses were removed as their responses would not be usable for the planned analyses. In these data cleaning steps, 893 (25%) of the 3510 initial cases were removed from the dataset, leading to a net sample size of 2617. This removal rate was equivalent among the four countries: Czech: 144/530 (27%), Italy: 249/1049 (24%), Netherlands: 295/1134 (26%), Sweden: 205/797 (26%), χ^2^(3) = 2.44, p = 0.486. Because of the multifaceted and mass-media communication recruitment strategy for this study, it is not possible to estimate the proportion of actual participants in this study out of the number of participants who were informed about and invited to participate in the study. Median duration of completing of the survey was 15 minutes, after data cleaning.

Table 1 presents the demographic characteristics of the participants. A high amount of demographic heterogeneity among the country samples can be observed. Noteworthy are the differences in residential urbanisation level; lifetime ecstasy use; and lifetime amphetamine use. It is important to acknowledge that these are samples taken from drug-using sub populations and should in no way be regarded as representative of the general population of any of the four countries. Remarkable is that the CAST sum score does not differ between the four samples (p = 0.55), nor does the main dependent variable of this study, the dichotomized CAST score (p = 0.36).

**Table 1:** Demographic statistics of the participants from the four countries.

	Combined (n = 2617)	Czech (n = 386)	Italy (n = 800)	Netherlands (n = 839)	Sweden (n = 592)	Tests	
Characteristic	M(SD) | n(%)	M(SD) | n(%)	M(SD) | n(%)	M(SD) | n(%)	M(SD) | n(%)	F(3,2613) | χ^2^(3)	*p*
Male	2009 (77%)	268 (69%)	630 (79%)	590 (70%)	521 (88%)	74.89	<0.0001
Age (years)	25.6 (8.53)	23.8 (8.30)	26.0 (7.92)	25.8 (8.70)	26.0 (9.08)	6.67	0.0002
Student	1119 (44%)	214 (56%)	428 (55%)	259 (33%)	218 (37%)	108.10	<0.0001
Res. Urbanisation level						244.00	<0.0001
City	757 (30%)	49 (13%)	266 (34%)	155 (20%)	287 (49%)		
Town	1278 (51%)	267 (70%)	404 (52%)	414 (53%)	193 (33%)		
Village	492 (19%)	64 (17%)	112 (14%)	214 (27%)	102 (18%)		
Living alone	420 (17%)	30 (8.0%)	97 (12%)	119 (15%)	174 (30%)	106.36	<0.0001
Unemployed	230 (9.1%)	20 (5.3%)	85 (11%)	71 (9.1%)	54 (9.3%)	9.67	0.022
Age at first cannabis use	16.4 (3.33)	15.5 (2.41)	16.1 (2.19)	16.2 (3.91)	17.5 (3.91)	36.80	<0.0001
Age at regular use	18.3 (4.73)	17.4 (3.53)	18.2 (4.27)	17.8 (4.98)	20.0 (5.38)	28.92	<0.0001
Number of use days (last year)						153.34	<0.0001
Incidentally (0–10 days)	691 (27%)	89 (23%)	141 (18%)	297 (35%)	164 (28%)		
Moderately (11–100 days)	791 (30%)	118 (31%)	199 (25%)	247 (29%)	227 (39%)		
Frequently (101–300 days)	645 (25%)	100 (26%)	263 (33%)	143 (17%)	139 (24%)		
(Almost) daily (>300 days)	480 (18%)	77 (20%)	194 (24%)	151 (18%)	58 (9.9%)		
Number of units per typical day	2.70 (2.35)	2.65 (2.14)	2.98 (2.40)	2.27 (2.14)	2.95 (2.59)	13.85	<0.0001
Cannabis use on weekdays	1242 (47%)	187 (48%)	519 (65%)	306 (36%)	230 (39%)	155.67	<0.0001
Cannabis use any time of day	804 (31%)	142 (37%)	298 (37%)	201 (24%)	163 (28%)	43.56	<0.0001
Amphetamine lifetime use	857 (33%)	126 (33%)	100 (13%)	456 (55%)	175 (30%)	327.50	<0.0001
Cocaine lifetime use	850 (33%)	80 (21%)	217 (27%)	424 (51%)	129 (22%)	189.29	<0.0001
Ecstacy lifetime use	983 (38%)	129 (34%)	113 (14%)	605 (73%)	136 (23%)	668.94	<0.0001
CAST sum score	5.79 (4.32)	5.74 (4.22)	5.88 (3.76)	5.63 (4.91)	5.93 (4.21)	0.70	0.553
CAST sum score> = 7	1058 (40%)	151 (39%)	328 (41%)	324 (39%)	255 (43%)	3.25	0.355

Note: Percentages are based on available cases (missing values omitted) Regular use is defined as use at least once per month; Number of use days is an aggregated version of the number of use days predictor used in the analysis, which has 12 levels; Lifetime use refers to any use/incidence of use; Cannabis use on weekdays refers to the question whether participants use cannabis in the weekends only, or also (or mainly) on working days; Presented in the proportion indicating that they use cannabis also/mainly on weekdays; Cannabis use any time of day refers to the question whether participants use cannabis on a specific time of the day or not; Presented is the proportion indicating that they use cannabis on any time of the day; Sample sizes of the four countries (and combined) are lower than the indicated number for some variables due to item missingness. F tests and chi-squared tests were performed to test the difference between the four countries. Samples are not representative of the general population in any of the four countries.

### CAST dimensionality and internal consistency

For the four countries, CAST's internal consistency and dimensionality were calculated. For the dimensionality, factors were extracted using exploratory factor analysis. The number of factors selected was based on the Kaiser criterion (eigenvalues>1). Internal consistency was estimated using Cronbach's α. For the Netherlands, internal consistency was good at Cronbach's α = 0.81. There was one factor with an eigenvalue above 1 (λ = 3.18). The 1-factor solution accounted for 55% of the variance. For Sweden, internal consistency was good at Cronbach's α = 0.74. There was one factor with an eigenvalue above 1 (λ = 2.74). The 1-factor solution accounted for 50% of the variance. For Czech Republic, internal consistency was good at Cronbach's α = 0.73. There were two factors with an eigenvalue above 1 (λ_1_ = 2.61; λ_2_ = 1.09). The 1-factor solution accounted for 47% of the variance, the 2-factor solution accounted for 64% of the variance. For Italy, internal consistency was acceptable at Cronbach's α = 0.63. There were two factors with an eigenvalue above 1 (λ_1_ = 2.13; λ_2_ = 1.17). The 1-factor solution accounted for 47% of the variance, the 2-factor solution accounted for 64% of the variance.

### Bivariate analysis

The 59 variables included in this first analysis step were related to the following categories:

1. Demographics of the participant: (gender, age, living alone, employment status, student status, urbanisation level of area of residence;
2. Cannabis use history: age at first ever use, age at first regular use, number of use days in the last 12 months, number of use days in the last 30 days;
3. Current cannabis use pattern: whether they predominantly use marihuana, hashish or both, whether they use mainly in the evening, mainly during daytime, or anytime, whether they use mostly in the weekends, or (also) during weekdays, how much they use on a typical day, how much they have used on the last occasion, and whether or not they have used cannabis together with others or alone on that occasion;
4. Acquisition of cannabis: whether they buy their own cannabis, grow their own cannabis, how much time it takes them to obtain cannabis, whether or not it is difficult for them to obtain cannabis within 24 hours, how often they have bought cannabis in the last 30 days, whether or not their dealer also offers them other substances besides cannabis;
5. Police contact in the last 12 months (drug law enforcement): whether they were stopped and searched by police, whether drugs (including cannabis) were found on them at that time, or on any other occasion;
6. Use of other substances: lifetime, last year, and last month use of alcohol, amphetamines, cocaine, ecstasy, GHB (gamma-hydroxybutyric acid), heroin, ketamine, or synthetic cannabinoids.

All variables were entered in the logistic regression analysis separately, with an intercept, and with the dichotomous variable CAST sum score ≥7 (Y/N) as the dependent variable. Analyses were performed on the training dataset (n = 2074). 36 variables were found to be associated with CAST ≥7 with p≤0.001. These 36 variables were transferred to the multivariate analysis step.

### Multivariate analysis

In the multivariate procedure, the 36 variables with a p≤0.001 association with CAST ≥7 were iteratively combined into 35,000 models consisting of between one and seven variables. Each of these models were fit using the training data and both Nagelkerke R^2^ and Akaike Information Criterion (AIC) goodness-of-fit statistics were calculated. Table 2 presents the 10 models with the highest Nagelkerke R^2^ goodness-of-fit values. Based on Nagelkerke R^2^, the best fitted 10 models were selected. The 19 different predictor variables from these 10 models were used for the recursive partitioning procedure. The predictor variables obtained from these models were:

**Table 2:** Ten models with optimal fit.

Model	Nagelkerke R^2^	AIC
CAST7∼freq_12mo+quant_typical_day+last_use_alone+coke_LT+sex+age_first_use	0.420	1805
CAST7∼freq_12mo+quant_last_day+last_use_alone+coke_LT+spice_LT+coke_LY	0.412	2034
CAST7∼freq_12mo+use_anytime+last_use_alone+buy_my_own+amph_LT+police_found_12mo	0.410	1960
CAST7∼freq_12mo+quant_typical_day+last_use_alone+coke_LT+spice_LY+ketam_LT	0.408	1833
CAST7∼freq_12mo+use_weekdays+quant_typical_day+last_use_alone+coke_LT	0.408	1833
CAST7∼freq_12mo+quant_last_day+last_use_alone+sex+age_first_use+spice_LY	0.407	2038
CAST7∼freq_12mo+last_use_alone+coke_LT+weed_user+age_first_use+unemployed	0.407	1974
CAST7∼freq_12mo+quant_typical_day+last_use_alone+sex+amph_LY	0.404	1840
CAST7∼freq_12mo+quant_typical_day+last_use_alone+amph_LT+spice_LY	0.404	1841
CAST7∼freq_12mo+quant_typical_day+last_use_alone+police_found_can_12mo+amph_LY	0.404	1841

Note: Variables correspond to those described in the section ‘Bivariate analysis’. AIC is Akaike Information Criterion, Nagelkerke R^2^ provides a goodness-of-fit index between 0–1.

1. Demographics (2 of 6 variables; 33%): gender (sex), being unemployed (unemployed);
2. Cannabis use history (2 of 4 variables; 50%): number of use days in the last 12 months (freq_12mo), age at first use (age_first_use);
3. Current cannabis use pattern (6 of 11; 55%): how much they use on a typical day (quant_typical_day), use on any time of the day (use_anytime), whether they used cannabis together with others or alone on the last occasion (last_use_alone), how much they used the last day they used cannabis (quant_last_day), mainly using weed (weed_user), use of cannabis during weekdays (use_weekdays);
4. Acquisition of cannabis (1 of 6; 17%): buy their own cannabis (buy_my_own);
5. Police contact in the last 12 months (1 of 3; 33%): cannabis found by the police during last year (police_found_can_12mo);
6. Use of other substances (7 of 24; 29%): lifetime amphetamine use (amph_LT), last year use of amphetamines (amph_LY), lifetime use of cocaine (coke_LT), last year use of cocaine (coke_LY), lifetime use of ketamine (ketam_LT), lifetime use of spice or other synthetic cannabinoids (spice_LT), last year use of spice or other synthetic cannabinoids (spice_LY).

### Recursive partitioning

**Figure 1:**
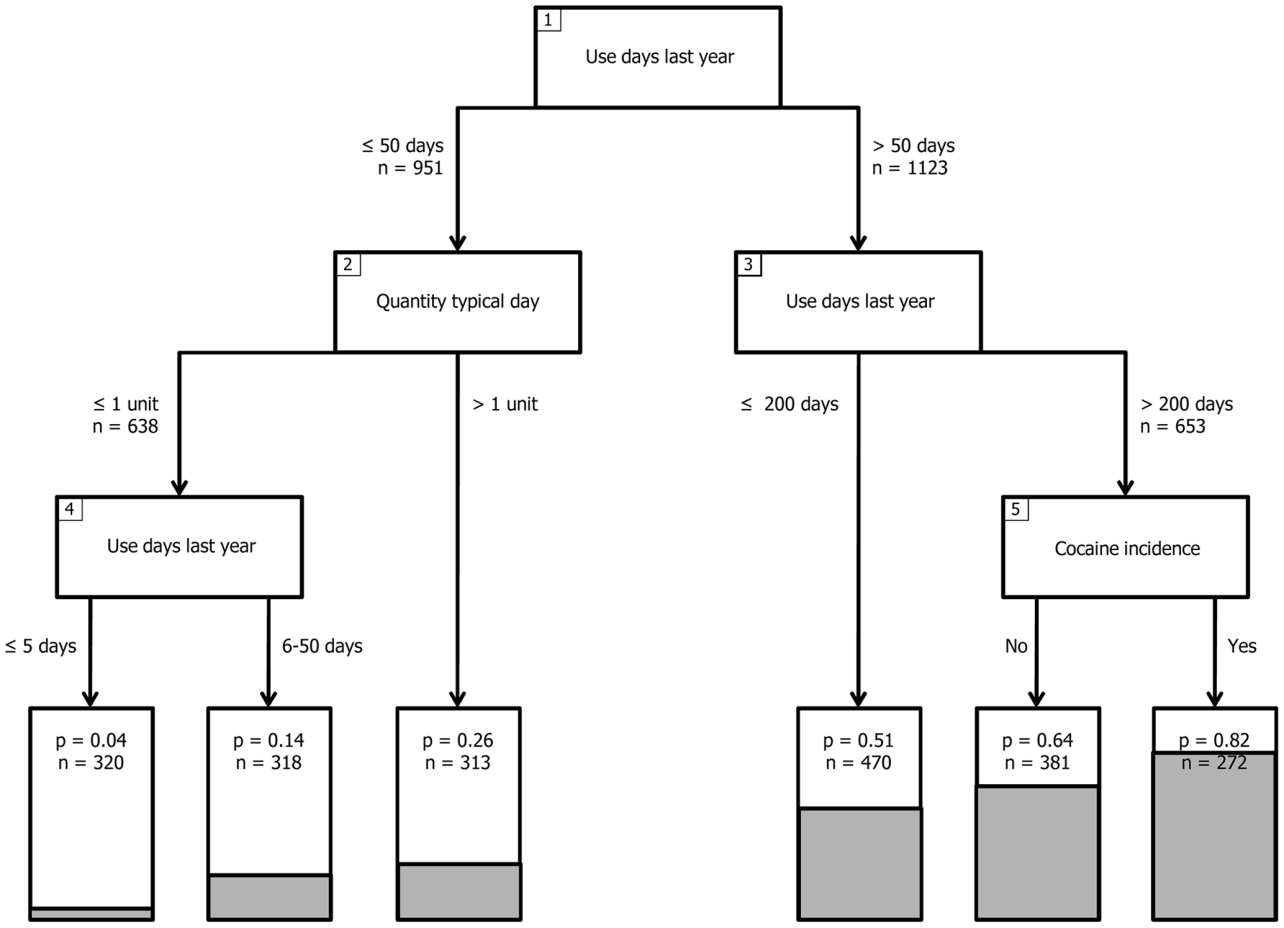
Recursive partitioning classification tree analysis of probability CAST ≥7 for four countries. Note: Generic classification model based on training data (n = 2074). The variables in the numbered boxes indicate the splitting variables identified in the recursive partitioning analysis. The cut-off value for each split, and the number of participants involved in each split is indicated next to the arrows diverting participants from the splitting variable. The six grey area's in the bottom of the lowest boxes (“terminal nodes”), and the “p” in these boxes indicates the *proportion* of participants in each partitioned area with scores of 7 or higher on the CAST. “n” in the lowest boxes indicates the number of participants in each of the terminal nodes. For each of the 5 splits, *p*<0.001.

Using the training dataset, the recursive partitioning procedure led to the model presented in Figure 1. In the numbered boxes of this figure, the splitting variables are presented. Those are the variables selected during recursive partitioning from the variables selected after multivariate analyses. The number of cannabis use days in the previous 12 months was the most influential predictor of CAST ≥7 in the classification tree. All selected variables are substance use related, and four of the five splits are made based on cannabis use variables - category (2) and (3). The lowest probability of CAST ≥7 (<10%) is found in participants reporting cannabis use on 50 days or less in the last 12 months, and who use one consumption unit (joint) or less on a typical use day. A high probability of CAST ≥7 (>60%) is found in participants reporting cannabis use on more than 200 days in the last 12 months. A little over 40% (272 of the 653) of this high risk subsample reports lifetime cocaine use, and those reporting lifetime cocaine use are at an even more elevated risk of CAST ≥7 (>80%).

### Validation of classification model

In order to estimate the predictive validity and accuracy of the proposed classification model, a number of classification performance statistics have been calculated by applying the model from Figure 1 to the training, validation, full, and the country-specific datasets (Table 3). Accuracy of the model using the training data seems higher than when using the validation data, although this difference is not significant: 0.73 vs. 0.69, χ^2^(1) = 3.21, p = 0.07 (2-sided). Classification using the generic model is superior to the no information rate for all analysed datasets. Accuracy of the generic model is highest for the Netherlands, followed by Sweden, Czech Republic, and Italy. The specificity of the model for classifying the participants from Czech Republic and Italy is relatively low.

**Table 3:** Performance statistics of the generic classification tree model.

Dataset	*n*	Accuracy (95% CI)	No Information Rate	*p* [Acc> NIR]	Sensitivity	Specificity	Positive Predictive Value	Negative Predictive Value
Training	2074	0.73 (0.71–0.75)	0.59	<0.0001	0.83	0.66	0.63	0.85
Validation	543	0.69 (0.65–0.73)	0.60	<0.0001	0.80	0.62	0.58	0.82
Full	2617	0.72 (0.70–0.74)	0.60	<0.0001	0.83	0.65	0.62	0.85
Czech	386	0.67 (0.62–0.72)	0.61	0.0067	0.85	0.55	0.55	0.86
Italy	800	0.66 (0.62–0.69)	0.59	<0.0001	0.90	0.49	0.55	0.87
Netherlands	839	0.80 (0.77–0.83)	0.61	<0.0001	0.82	0.79	0.71	0.87
Sweden	592	0.73 (0.70–0.77)	0.57	<0.0001	0.74	0.73	0.67	0.79

Note: Accuracy indicates the proportion of correctly classified cases, with associated confidence interval; No information rate (NIR) is 1-[proportion of CAST≥7] in the sample; Difference between model accuracy and NIR tested using a one-sided binomial test; Sensitivity = [number of correctly classified CAST ≥7]/[number of CAST ≥7 in sample]; Specificity = [number of correctly classified CAST <7]/[number of CAST <7 in sample]; Positive predictive value = [number of correctly classified CAST ≥7]/[all classified CAST ≥7]; Negative predictive value = [number of correctly classified CAST <7]/[all classified CAST <7]. The country datasets contain both participants from the training and the validation dataset.

### Country-specific tree models

As can be inferred from Table 1, the presented country-level averages and category distributions for the four countries show a high degree of heterogeneity. Table 3 shows that participants from Czech Republic and Italy are less accurately classified by the generic model. Therefore, it is explored to what extent country-specific models outperform the generic model with regard to the performance statistics presented in Table 3. Therefore, we have repeated the recursive partitioning analysis with the same set of 19 variables (Table 2), but now the country datasets have been partitioned separately.

**Figure 2:**
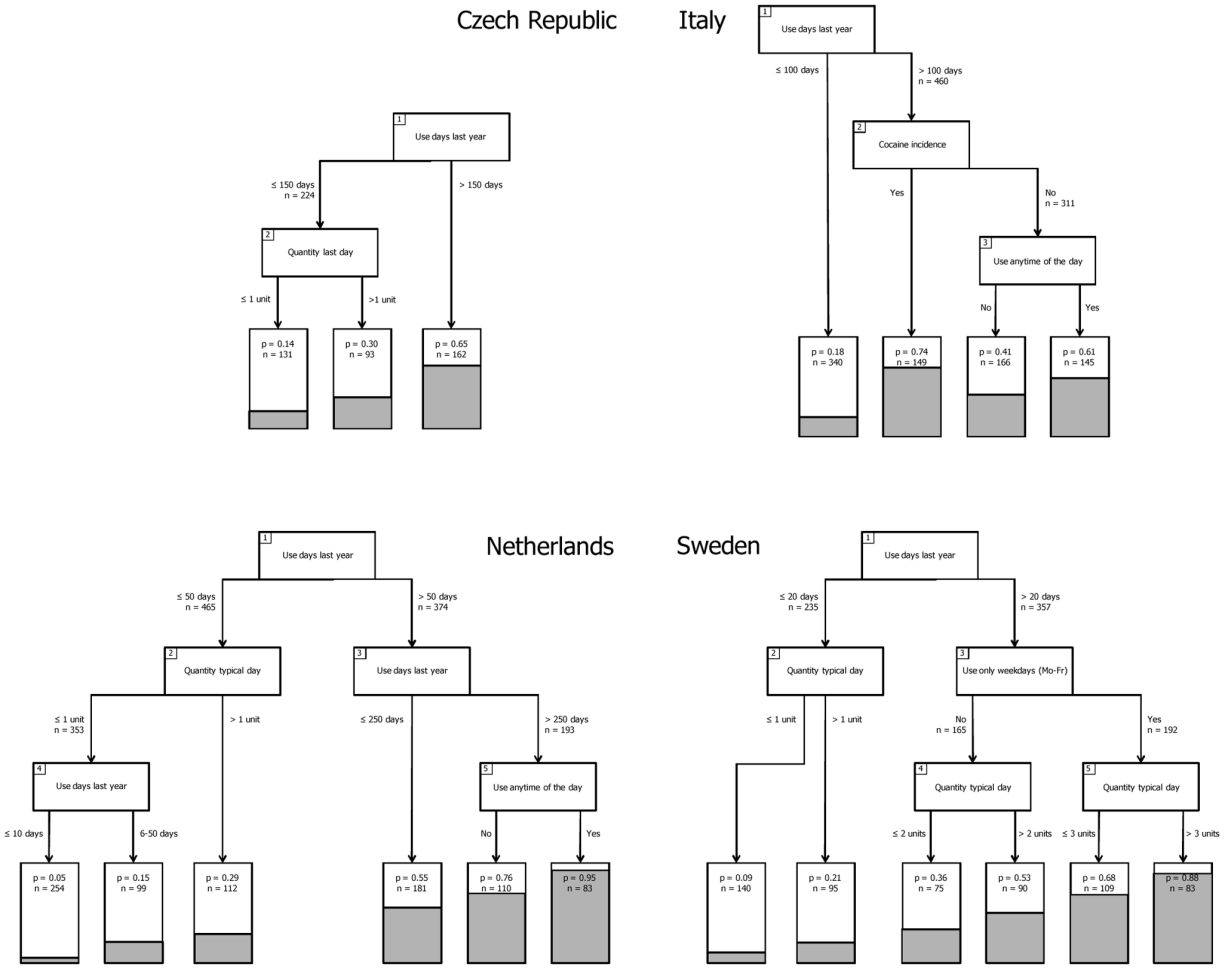
Country specific classification tree models. Note: Country specific classification tree models for the Czech Republic (top-left), Italy (top-right), Netherlands (bottom-left) and Sweden (bottom-right). The variables in the numbered boxes indicate the splitting variables identified in the recursive partitioning analysis. The cut-off value for each split, and the number of participants involved in each split is indicated next to the arrows diverting participants from the splitting variable. The six grey area's in the bottom of the lowest boxes (“terminal nodes”), and the “p” in these boxes indicates the proportion of participants in each partitioned area with scores of 7 or higher on the CAST. “n” in the lowest boxes indicates the number of participants in each of the terminal nodes.

Figure 2 shows that many of the same variables that were selected for the generic model are selected for the country specific models. The ‘new’ variables in the country specific models are related to *when* participants use in Sweden (also during weekdays Y/N) and in the Netherlands (any time of the day Y/N). In the Czech, Netherlands, and Swedish model, cocaine incidence was found to be insignificant. In Czech Republic, the variable “quantity on the last day” was selected as a splitting variable in the model. This variable is strongly correlated with (and quite similar to) the variable “quantity on a typical day” in the generic, the Netherlands, and the Swedish model (Pearson r = 0.69, t(2362) = 46.1, p<0.001). However, the order and number of variables selected, and the cut-off values for the variables in the country-specific models differ from those in the generic model.

The performance statistics (Table 4) indicate that by using country specific models the classification of participants from Italy improves significantly (p = 0.01) and the classification of participants from Czech Republic improves marginally (p = 0.08). The specificity of the Italian and Czech model has improved as well in comparison to the generic model. The country specific models for the Netherlands and Sweden do not lead to significantly improved classification accuracy. Overall, confidence intervals of the classification accuracy using the country specific models for the four countries overlap, justifying the conclusion that none of the country specific models outperforms any of the others.

**Table 4:** Country specific classification tree models compared to the generic tree model.

Dataset	*n*	Country Specific Model Accuracy (95% CI)	Generic Model Accuracy (95% CI)	χ^2^(1)	*p* [Acc> GMA]	Sensitivity	Specificity	Positive Predictive Value	Negative Predictive Value
Czech	386	0.73 (0.69–0.78)	0.67 (0.62–0.72)	2.99	0.08	0.70	0.76	0.65	0.79
Italy	800	0.72 (0.69–0.75)	0.66 (0.62–0.69)	6.45	0.01	0.61	0.80	0.68	0.75
Netherlands	839	0.80 (0.77–0.82)	0.80 (0.77–0.83)	0.00	1.00	0.81	0.77	0.71	0.87
Sweden	592	0.75 (0.71–0.79)	0.73 (0.70–0.77)	0.53	0.47	0.76	0.74	0.69	0.81

Note: Country Specific Model refers to the classification trees presented in Figure 2; Generic Model refers to the overarching model presented in Figure 1; Accuracy indicates the proportion of correctly classified cases, with associated confidence interval; Difference in accuracy between Country Specific Model and Generic Model was tested using a 2-sided Chi-Square test; Sensitivity = [number of correctly classified CAST ≥7]/[number of CAST ≥7 in sample]; Specificity = [number of correctly classified CAST <7]/[number of CAST <7 in sample]; Positive predictive value = [number of correctly classified CAST ≥7]/[all classified CAST ≥7]; Negative predictive value = [number of correctly classified CAST <7]/[all classified CAST <7]. The country datasets contain both participants from the training and the validation dataset.

## Discussion

### Main conclusions

Of the 59 variables included in step 1 of the presented analysis, three variables may be sufficient to construct a generic model to classify cannabis users from Czech Republic, Italy, the Netherlands and Sweden with 65–73% accuracy in groups of above or below a CAST cut-off score, indicative of meeting the criteria for cannabis use disorder formulated in DSM-V. Using three additional variables, four country-specific models have been constructed, leading to (marginally) significant improvement in classification accuracy of cannabis users from Czech Republic and Italy. All six variables except one (lifetime cocaine use) are associated with cannabis use in the last year – as one would expect considering the focus of items from the CAST on cannabis use. The number of cannabis use days in the last 12 months is the most dominant predictor of CAST score below 7, or 7 and up. Apparently, 12-month estimates are more predictive of cannabis use problems than 30-day estimates, which were also available in our data.

What is interesting from a qualitative comparison between the Swedish and the Netherlands' country specific model, is how similar the two models are. Sweden and the Netherlands have very different cannabis control policies. In the Netherlands the sale, possession and use of small quantities of cannabis is tolerated. In Sweden, cannabis use and possession is treated on the same level as other illegal substances. Drug felonies may lead to several years of imprisonment, although in practise, courts sentence out lower penalties for the sale of cannabis than for example for amphetamine or heroin [49].

Although separate country models outperform generic models for at least one of the four countries (Italy, p = 0.01), differences between the models mainly exist in the cut-off values of variables and not so much in the selected variables themselves. This may be perceived as supportive of the generalizability of the proposed model, although the generalizability of the model should preferably be tested using newly collected data. On the other hand, the differences in the cut-off values especially in the variables representing the number of use days may be of specific interest.

In order to find the proposed model, a data-driven and explorative instead of a hypothesis driven approach was chosen. Whether or not to account for multiple comparisons by adjusting the α level while exploring data for potential predictors is a matter of debate. Based on work by Bendel & Affifi [50] on stepwise regression, a p≤0.05 level may already exclude important variables from the model. Although a higher p-value limit raises the risk of Type I error, it reduces the risk of not finding a relationship between variables that is really there (Type II error), i.e., it improves statistical power. However, considering the fact that the sample size in this study is relatively large, it is not likely that a shortage of power will lead to Type II error. Therefore, application of Bonferroni correction for the analyses seemed justified.

### Limitations

The results of this study should be considered in light of its limitations. First and foremost, the representativeness of the samples is a matter of debate. Although the purpose of the study has not been to include a representative sample of a circumscribed population of cannabis, an assumption underlying the presentation of the results as potentially generalizable to a wider cannabis using population is that the associations between variables in this study would also have been found in a representative sample of cannabis users from the four countries. If misrepresentations in the recruited samples have led to reporting invalid associations, it is not possible to estimate the direction of this error a priori; that is, it is not known whether associations may in reality be stronger or weaker than reported. Although this study is not unique in having this limitation, it may have influenced its results and conclusions to an indeterminable amount.

Another limitation to this study is that the CAST which was used for the operationalization of at-risk cannabis use, is not a gold reference for at-risk use. Although multiple psychometric studies in European countries have demonstrated the favourable validity and reliability of the CAST in comparison to diagnostic interviews and other cannabis screening tests, the variables presented in this paper as associated with at-risk cannabis use, are possibly only associated with lower or higher CAST scores. This possibility is more than theoretical considering the fact that some of the CAST items bear similarity with the factors we have entered in the analyses as potential predictors. To give an example, the CAST item “Have you smoked cannabis when you were alone” is quite literally the same as the question whether or not they used cannabis together with others or alone on during the last occasion of use. In addition, the factorial structure may not be identical nor may the cut-off value of 7 be optimal in all cultures. An extensive cross-cultural validation of the CAST would therefore be desirable. On the other hand, it is noteworthy that among the four countries the proportion of respondents with a score of 7 or more on the CAST is almost equal (Table 1).

Recursive partitioning is primarily a data driven approach. Debate remains as to whether recursive partitioning is prone to overfitting data or not. Either way, the resulting classification tree is always one of the possible solutions rather than the only solution to fit the data. Another common critique on recursive partitioning is its sensitivity to small changes in the data used. The stability of the presented model is evaluated using cross-validation: the full dataset was split into a training and a validation set; the analysis of these two sets led to comparable results. A methodologically stronger approach would have been to use two separately collected datasets, the first to construct the classification tree, and the second to evaluate the model and to calculate the performance statistics. Therefore a validation of the model in a new sample would be desirable before future use of the presented model is considered.

A final potential limitation is the variation in sample sizes between the countries. In general, a larger sample size may provide stronger statistical support for a larger tree, with more branches. This also seems to be the case in the current analysis, in which the tree for the Czech Republic (n = 386) consists of fewer branches than the other trees. The larger sample sizes in some countries do not per se make the generic model fit these countries better than the others. It is possible that the overall model fit reflects a comparable cannabis using culture in the Netherlands and Sweden, while cannabis use cultures in Italy and the Czech Republic are more different.

### Strengths

A strength of the current study is the unique multi-country dataset, containing data from a total of 2617 cannabis users from four countries. Two of these countries are known to have a relatively liberal (Czech Republic, the Netherlands) cannabis policy, while in the two others cannabis policy and enforcement tends to be more strict (Sweden, Italy). Another strength of the study is the three-step statistical approach in finding the optimal set of predictors – an approach which allowed us initially to include a large number of potential predictors. The resulting performance statistics derived from the models (sensitivity/specificity) indicate that the proposed model may actually be useful in practise as a screener for potential cannabis use disorder in a population of current cannabis users, in addition to the CAST.

### Implications

If the performance statistics of the model can be replicated using additional data, our results may have implications for preventive interventions, and for prevention policy. Presumably, a common goal of public health interventions addressed at cannabis use is to reduce the number of users that develop a problematic use pattern or dependence, based on the model proposed in this paper, if cannabis use is limited to weekly use or less (max. 50 use days per year, see Figure 1), the risk across individuals of developing use disorders is less than 20% in the studied population (with an average rate of 40%). If this finding can be replicated, it provides a quantification of the general notion that more consumption leads to a higher risk on dependence. In addition, this statistically derived finding that the number of use days and the quantity of use per occasion best predicts cannabis related problems as indicated by the CAST confirms the position of heavy use being a good predictor and explaining addictive disorders, as was proposed in a recent debate by Rehm and colleagues [51], [52]. In this debate, the authors provided evidence to redefine the concept of substance use disorders in terms of heavy use over time, and listed a number of advantages including destigmatisation and initiation of lifestyle interventions that could follow from such a conceptual redefinition.

## Conclusions

This study demonstrated how demographic, drug use, cannabis acquisition and drug market variables can be used to construct classification trees using recursive partitioning. The classification trees indicate an individual's probability of being an at-risk cannabis user. The characteristics associated most strongly with problematic cannabis use in the generic model are the number of use days and quantity of cannabis consumed per use day. Also lifetime cocaine use status seems strongly associated. In the country-specific models for Czech Republic, Netherlands, and Swedish model however, cocaine incidence was found to be insignificant. The cut-off values of the classification variables varied among the countries, most notably the number of cannabis use days per year. Although the country specific models fitted the data somewhat better, and significantly better for one country (Italy), it was feasible to propose a single classification tree with acceptable performance to classify cannabis users in groups with low or high risk of meeting DSM-V criteria for cannabis use disorder.

## References

[1] European Monitoring Centre for Drugs and Drug Addiction (EMCDDA) (2013) Perspectives on Drugs: Characteristics of frequent and high-risk cannabis users. Lisbon: EMCDDA.

[2] van der Pol P, Liebregts N, de Graaf R, Korf DJ, van den Brink W, et al (2013) Predicting the transition from frequent cannabis use to cannabis dependence: A three-year prospective study. Drug Alcohol Depend 133: 352–359. 23886472 10.1016/j.drugalcdep.2013.06.009

[3] Hammersley R, Leon V (2006) Patterns of cannabis use and positive and negative experiences of use amongst university students. Addict Res Theory 14: 189–205.

[4] Coffey C, Carlin JB, Lynskey M, Li N, Patton GC (2003) Adolescent precursors of cannabis dependence: findings from the Victorian Adolescent Health Cohort Study. Br J Psychiatry 182: 330–336. 12668409 10.1192/bjp.182.4.330

[5] Legleye S, Piontek D, Kraus L, Morand E, Falissard B (2013) A validation of the Cannabis Abuse Screening Test (CAST) using a latent class analysis of the DSM-IV among adolescents. Int J Methods Psychiatr Res 22: 16–26. 23519957 10.1002/mpr.1378 PMC6878590

[6] Gossop M, Darke S, Griffiths P, Hando J, Powis B, et al (1995) The Severity of Dependence Scale (SDS): psychometric properties of the SDS in English and Australian samples of heroin, cocaine and amphetamine users. Addiction 90: 607–614. 7795497 10.1046/j.1360-0443.1995.9056072.x

[7] Martin G, Copeland J, Gates P, Gilmour S (2006) The Severity of Dependence Scale (SDS) in an adolescent population of cannabis users: reliability, validity and diagnostic cut-off. Drug Alcohol Depend 83: 90–93. 16310973 10.1016/j.drugalcdep.2005.10.014

[8] Adamson SJ, Sellman JD (2003) A prototype screening instrument for cannabis use disorder: the Cannabis Use Disorders Identification Test (CUDIT) in an alcohol dependent clinical sample. Drug Alcohol Rev 22: 309–315. 15385225 10.1080/0959523031000154454

[9] Annaheim B, Rehm J, Gmel G (2008) How to screen for problematic cannabis use in population surveys: an evaluation of the Cannabis Use Disorders Identification Test (CUDIT) in a Swiss sample of adolescents and young Adults. Eur Addict Res 14: 190–197. 18583916 10.1159/000141643

[10] Legleye S, Karila L, Beck F, Reynaud M (2007) Validation of the CAST, a general population Cannabis Abuse Screening Test. Journal of substance use 12: 233–242.

[11] Annaheim B (2013) Who is smoking pot for fun and who is not? An overview of instruments to screen for cannabis-related problems in general population surveys. Addict Res Theory 21: 410–428.

[12] Hibell B, Guttormsson U, Ahlström S, Balakireva O, Bjarnason T, et al.. (2012) The 2011 ESPAD Report: Substance Use Among Students in 36 European Countries. Stockholm: The Swedish Council for Information on Alcohol and Other Drugs.

[13] Bastiani L, Siciliano V, Curzio O, LuppiC, Gori M, et al (2013) Optimal scaling of the CAST and of SDS Scale in a national sample of adolescents. Addict Behav 38: 2060–2067. 23396173 10.1016/j.addbeh.2012.12.016

[14] Cuenca-Royo AM, Sánchez-Niubó A, Forero CG, Torrens M, Suelves JM, et al (2012) Psychometric properties of the CAST and SDS scales in young adult cannabis users. Addict Behav 37: 709–715. 22386300 10.1016/j.addbeh.2012.02.012

[15] Thanki D, Domingo-Salvany A, Anta GB, Mañez AS, Aleixandre NL, et al (2013) The Choice of Screening Instrument Matters: The Case of Problematic Cannabis Use Screening in Spanish Population of Adolescents. ISRN Addict 2013: 723131. 25969832 10.1155/2013/723131 PMC4392974

[16] Legleye S, Piontek D, Kraus L (2011) Psychometric properties of the Cannabis Abuse Screening Test (CAST) in a French sample of adolescents. Drug Alcohol Depend 113: 229–235. 20869178 10.1016/j.drugalcdep.2010.08.011

[17] Verweij KJ, Zietsch BP, Lynskey MT, Medland SE, Neale MC, et al (2010) Genetic and environmental influences on cannabis use initiation and problematic use: a meta-analysis of twin studies. Addiction 105: 417–430. 20402985 10.1111/j.1360-0443.2009.02831.x PMC2858354

[18] Hyman SM, Sinha R (2009) Stress-related factors in cannabis use and misuse: implications for prevention and treatment. J Subst Abuse Treat 36: 400–413. 19004601 10.1016/j.jsat.2008.08.005 PMC2696937

[19] Tziraki S (2012) Mental disorders and neuropsychological impairment related to chronic use of cannabis. Rev Neurol 54: 750–760. 22673951

[20] Rey JM, Martin A, Krabman P (2004) Is the party over? Cannabis and juvenile psychiatric disorder: the past 10 years. J Am Acad Child Adolesc Psychiatry 43: 1194–1205. 15381886 10.1097/01.chi.0000135623.12843.60

[21] Large M, Sharma S, Compton MT, Slade T, Nielssen O (2011) Cannabis use and earlier onset of psychosis: a systematic meta-analysis. Arch Gen Psychiatry 68: 555–561. 21300939 10.1001/archgenpsychiatry.2011.5

[22] Fernandez-Espejo E, Viveros MP, Núñez L, Ellenbroek BA, Rodriguez de Fonseca F (2009) Role of cannabis and endocannabinoids in the genesis of schizophrenia. Psychopharmacology (Berl) 206: 531–549. 19629449 10.1007/s00213-009-1612-6

[23] Martín-Santos R, Fagundo AB, Crippa JA, Atakan Z, Bhattacharyya S, et al (2010) Neuroimaging in cannabis use: a systematic review of the literature. Psychol Med 40: 383–398. 19627647 10.1017/S0033291709990729

[24] Verdejo-García A, Pérez-García M, Sánchez-Barrera M, Rodriguez-Fernández A, Gómez-Río M (2007) Neuroimaging and drug addiction: Neuroanatomical correlates of cocaine, opiates, cannabis and ecstasy abuse. Rev Neurol 44: 432–439. 17420970

[25] Daniel JZ, Hickman M, Macleod J, Wiles N, Lingford-Hughes A, et al (2009) Is socioeconomic status in early life associated with drug use? A systematic review of the evidence. Drug Alcohol Rev 28: 142–153. 19320699 10.1111/j.1465-3362.2008.00042.x

[26] Chen CY, Storr CL, Anthony JC (2009) Early-onset drug use and risk for drug dependence problems. Addict Behav 34: 319–322. 19022584 10.1016/j.addbeh.2008.10.021 PMC2677076

[27] Looby A, Earleywine M (2007) Negative consequences associated with dependence in daily cannabis users. Subst Abuse Treat Prev Policy 2: 3. 17214886 10.1186/1747-597X-2-3 PMC1783648

[28] Moss HB, Chen CM, Yi HY (2012) Measures of substance consumption among substance users, DSM-IV abusers, and those with DSM-IV dependence disorders in a nationally representative sample. J Stud Alcohol Drugs 73: 820–828. 22846246 10.15288/jsad.2012.73.820 PMC3410949

[29] Walden N, Earleywine M (2008) How high: quantity as a predictor of cannabis-related problems. Harm Reduct J 5: 20. 18510745 10.1186/1477-7517-5-20 PMC2438353

[30] Zeisser C, Thompson K, Stockwell T, Duff C, Chow C, et al (2011) A ‘standard joint’? The role of quantity in predicting cannabis-related problems. Addict Res Theory 20: 82–92.

[31] van der Pol P, Liebregts N, de Graaf R, Ten Have M, Korf DJ, et al (2013) Mental health differences between frequent cannabis users with and without dependence and the general population. Addiction 108: 1459–1469. 23530710 10.1111/add.12196

[32] van den Bree MB, Pickworth WB (2005) Risk factors predicting changes in marijuana involvement in teenagers. Arch Gen Psychiatry 62: 311–319. 15753244 10.1001/archpsyc.62.3.311

[33] Kerr WT, Lau EP, Owens GE, Trefler A (2012) The future of medical diagnostics: large digitized databases. Yale J Biol Med 85: 363–377. 23012584 PMC3447200

[34] Mello MM, Messing NA (2011) Restrictions on the use of prescribing data for drug promotion. N Engl J Med 365: 1248–1254. 21812664 10.1056/NEJMhle1107678

[35] Hellemann G, Conner B, Anglin MD, Longshore D (2009) Seeing the trees despite the forest: Applying recursive partitioning to the evaluation of drug treatment retention. J Subst Abuse Treat 36: 59–64. 18599252 10.1016/j.jsat.2008.03.005

[36] Yonkers KA, Gotman N, Kershaw T, Forray A, Howell HB, et al (2010) Screening for Prenatal Substance Use: Development of the Substance Use Risk Profile-Pregnancy Scale. Obstet Gynecol 116: 827–833. 20859145 10.1097/AOG.0b013e3181ed8290 PMC3103106

[37] Blankers M, Koeter MWJ, Schippers GM (2013) Baseline predictors of treatment outcome in Internet-based alcohol interventions: a recursive partitioning analysis alongside a randomized trial. BMC Public Health 13: 455. 23651767 10.1186/1471-2458-13-455 PMC3662562

[38] Huang Y, Britton J, Hubbard R, Lewis S (2013) Who receives prescriptions for smoking cessation medications? An association rule mining analysis using a large primary care database. Tob Control 22: 274–279. 22246781 10.1136/tobaccocontrol-2011-050124

[39] Breiman L, Friedman J, Olshen R, Stone C (1984) Classification and Regression Trees. Belmont (CA): Wadsworth.

[40] Hawkins DM (1997) FIRM: Formal Inference-based Recursive Modeling, PC Version, Release 2.1 (Technical Report 546). Minnesota: University of Minnesota, School of Statistics.

[41] Strobl C, Malley J, Tutz G (2009) An introduction to recursive partitioning: rationale, application, and characteristics of classification and regression trees, bagging, and random forests. Psychol Methods 14: 323–348. 19968396 10.1037/a0016973 PMC2927982

[42] Trautmann F, Kilmer B, Turnbull P (Eds) (2013) Further insights into aspects of the EU illicit drugs market. Luxembourg: Publications Office of the European Union.

[43] Swan GE, Javitz HS, Jack LM, Curry SJ, McAfee T (2004) Heterogeneity in 12-month outcome among female and male smokers. Addiction 99: 237–250. 14756716 10.1111/j.1360-0443.2003.00629.x

[44] Nagelkerke NJD (1991) A note on a general definition of the coefficient of determination. Biometrika 78: 691–692.

[45] Zhang H, Singer B (1999) Recursive partitioning in the health sciences. New York: Springer Verlag.

[46] Hothorn T, Hornik K, Strobl C, Zeileis A (2013) party: A Laboratory for Recursive Partytioning. R package version 1.0-10 Available: http://cran.r-project.org/web/packages/party/index.html. Accessed 2013 November 28..

[47] R Core Team (2013) R: A language and environment for statistical computing. R Foundation for Statistical Computing, Vienna, Austria Available: http://www.R-project.org/. Accessed 2013 November 28..

[48] Therneau TM, Atkinson EJ (1997) An introduction to recursive partitioning using the RPART routines. Minnesota: Mayo Clinic.

[49] UNODC (2007) Sweden's successful drug policy: A review of the evidence. Available: http://www.unodc.org/pdf/research/Swedish_drug_control.pdf. Accessed 2013 November 28.

[50] Bendel RB, Afifi AA (1977) Comparison of stopping rules in forward regression. J Am Stat Ass 72: 46–53.

[51] Rehm J, Anderson P, Gual A, Kraus L, et al (2014) The tangible common denominator of substance use disorders: a reply to commentaries to Rehm et al. (2013a) [Letter to the Editor]. Alcohol Alcohol 49: 118–122. 10.1093/alcalc/agt171 24226811

[52] Rehm J, Marmet S, Anderson P, Gual A, Kraus L, et al (2013) Defining substance use disorders: do we really need more than heavy use? Alcohol and Alcohol 48: 633–640. 10.1093/alcalc/agt127 23926213

[53] EMCDDA (2008) A cannabis reader: global issues and local experiences, Monograph series 8, Volume 2, European Monitoring Centre for Drugs and Drug Addiction, Lisbon. p. 44.

[54] American Psychiatric Association (2013) Diagnostic and Statistical Manual of Mental Disorders. American Psychiatric Association, Arlington, VA

